# BV–*R_on,sp_* Trade-Off Optimization in a Floating-P-Island-Assisted Silicon Shielded-Gate Trench MOSFET

**DOI:** 10.3390/mi17090999

**Published:** 2026-08-24

**Authors:** Zequ Han, Juan Luo, Yunhao Deng, Zhi Lin, Yifei Duan, Shengdong Hu

**Affiliations:** School of Microelectronics and Communication Engineering, Chongqing University, Chongqing 401331, China; 202412131077t@stu.cqu.edu.cn (Z.H.); hushengdong@hotmail.com (S.H.)

**Keywords:** shielded-gate trench MOSFET, floating P-island, electric-field modulation, breakdown voltage, specific on-resistance, TCAD simulation

## Abstract

To address trench-bottom electric-field concentration and the limited BV–*R_on,sp_* trade-off of the conventional shielded-gate trench MOSFET (Conventional SGT), a floating-P-island-assisted silicon SGT MOSFET is proposed. Sentaurus TCAD is used to investigate the effects of increasing the number of floating P-islands from 0 to 3 on BV, *R_on,sp_*, FOM, and electric-field distribution. Within this range, the three-floating-P-island device (3FPI-SGT) provides a favorable BV–*R_on,sp_* trade-off; its drift-region doping matching, dynamic characteristics, and parameter sensitivity are further analyzed. Local depletion around multiple vertically discrete P-islands generates secondary electric-field peaks that share the potential drop with the main trench-bottom peak, reducing field crowding and improving voltage utilization of the drift region. After optimization, the maximum BV and FOM reach 177.7 V and 11.48 MW·cm^−2^, respectively. At an identical *R_on,sp_* of 2.62 mΩ·cm^2^, BV increases by 54.6%. At *V_DS_* = 80 V, *C_oss_* and *C_rss_* decrease by approximately 12.8% and 12.5%, while total switching energy increases by only approximately 2.5%. A clear BV advantage is retained under ±20% single-factor deviations in key P-island parameters. Thus, the proposed structure significantly improves the silicon SGT MOSFET BV–*R_on,sp_* trade-off with a limited dynamic-performance penalty.

## 1. Introduction

With the development of power electronic systems toward higher efficiency, higher power density, and higher operating frequency, low- and medium-voltage power MOSFETs are required not only to reduce conduction loss but also to balance gate-drive loss and switching speed. Trench-gate MOSFETs reduce the on-resistance per unit cell area by increasing channel density; shielded-gate trench (SGT) MOSFETs further introduce source-connected shield polysilicon inside the trench to weaken gate–drain coupling and reduce *C_rss_* and *Q_gd_*, and have therefore attracted extensive attention in synchronous rectification, DC–DC conversion, and low- and medium-voltage high-frequency power conversion [1,2,3]. However, while maintaining a low *R_on,sp_* and weak gate–drain coupling, the off-state blocking capability of the Conventional SGT remains limited by the electric-field distribution in the drift region.

For silicon vertical power MOSFETs, a fundamental trade-off between breakdown voltage (BV) and specific on-resistance (*R_on,sp_*) is imposed by the drift region: increasing BV generally requires reducing the drift-region doping concentration or increasing the epitaxial-layer thickness, which increases *R_on,sp_* [4,5,6,7]. In SGT devices, the shield electrode participates in depletion of the drift region; nevertheless, under high-voltage off-state conditions, local electric-field crowding can still occur at the trench bottom and in the adjacent silicon region, leaving the lower drift region underutilized for voltage blocking. To address this issue, electric-field modulation approaches including superjunctions, buried oppositely doped regions, high-k-dielectric-assisted structures, multiple epitaxial layers, shield layers, and vertically nonuniform doping profiles have been used to redistribute the potential drop in the drift region and improve device performance trade-offs [5,6,7,8,9,10,11,12,13,14,15,16,17,18,19,20]. These studies show that reshaping the electric-field distribution through local charge compensation or dielectric/doping engineering, rather than relying solely on lower drift-region doping or a thicker drift region, is an important route for further improving SGT performance.

Floating P-type regions represent another type of charge-modulation approach. Vaid and Padha introduced the floating-island concept into trench-gate power MOSFETs and analyzed its effects on the electric field and conduction characteristics [21]. Deng et al. further proposed a 150 V SGT-MOSFET with a floating P-pillar beneath the trench and systematically investigated its static and dynamic characteristics, with particular attention to transient capacitance and high-frequency dynamic *R_DS_*(on) [22]. These studies demonstrated the effectiveness of introducing floating P-type charge beneath the trench to modulate the drift-region electric field. Compared with previously reported floating P-pillars or specific floating-region structures, however, the effects of the number and spatial distribution of multiple vertically discrete floating P-islands, the coupling between increasing the number of islands and increasing the total P-type compensation charge, and the relationship between static gain and dynamic penalty under identical conduction-loss conditions have received relatively limited systematic discussion.

To address these issues, this work proposes a floating-P-island-assisted silicon shielded-gate trench MOSFET (FPI-SGT), in which multiple mutually separated floating P-islands are introduced vertically beneath the source-connected shield polysilicon. Unlike a single floating P-region or a continuous floating P-pillar, the P-type modulation region is divided into several vertically discrete islands. The local P/N junctions formed at different depths generate multiple secondary electric-field peaks in the off state, which share the potential drop with the main trench-bottom peak, thereby improving drift-region voltage utilization and the BV–*R_on,sp_* trade-off. Sentaurus TCAD is used to investigate the static performance and field-modulation mechanism of structures containing 0–3 floating P-islands. Comparisons at identical total P-type compensation charge, matched *R_on,sp_*, and identical drift-region doping are further performed to distinguish the effects of island number and spatial distribution, total compensation charge, and drift-region doping adjustment. Within the investigated range, the 3FPI-SGT is selected for detailed evaluation of dynamic characteristics and parameter-deviation sensitivity. The results indicate that multiple vertically discrete floating P-islands can markedly enhance the off-state blocking capability while maintaining low conduction loss and a limited dynamic-performance penalty, providing a new electric-field-modulation approach for improving the BV–*R_on,sp_* trade-off of silicon SGT MOSFETs.

## 2. Device Structure and Simulation Methods

**Figure 1:**
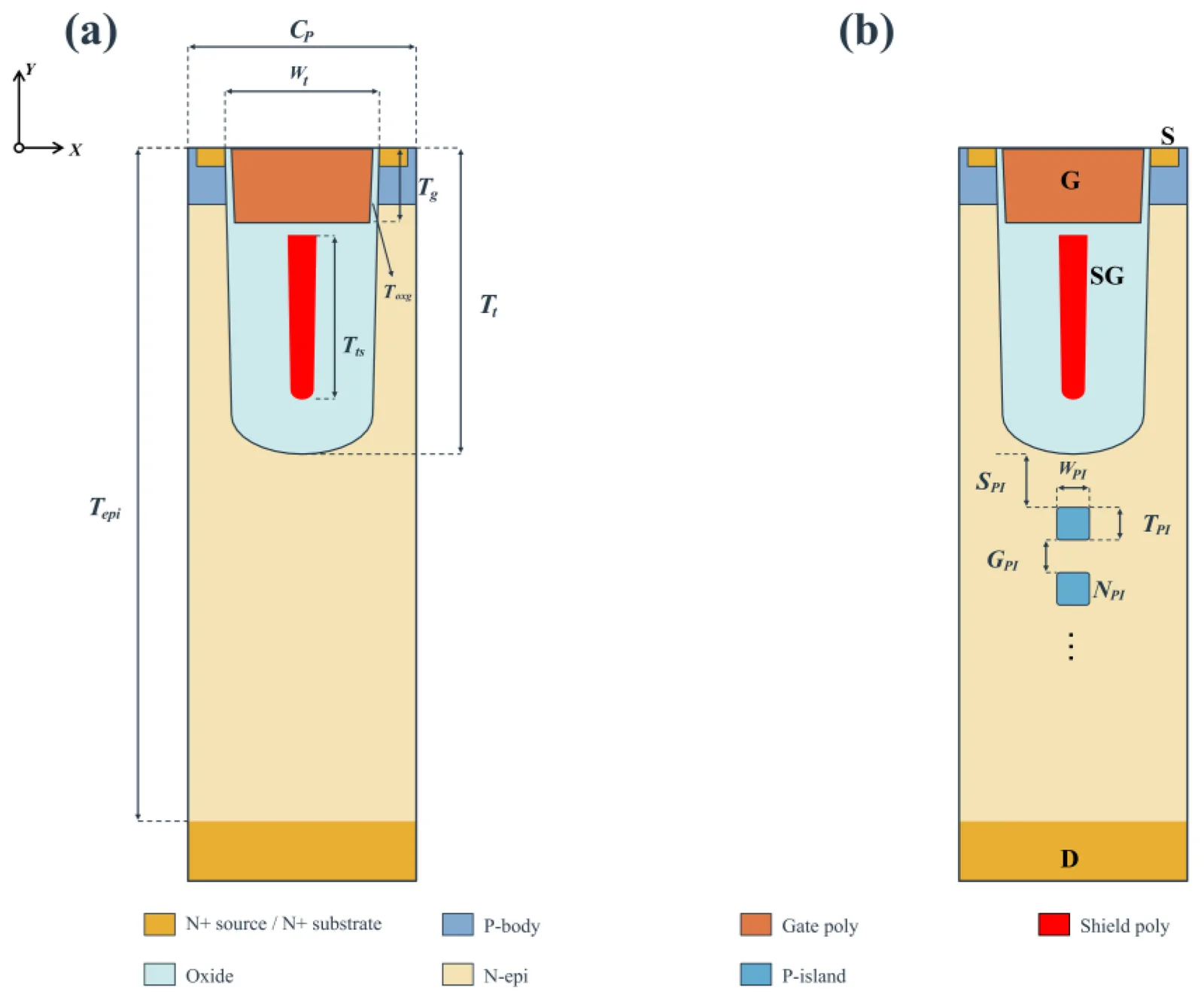
Schematic two-dimensional cross-sectional structures of the Conventional SGT and FPI-SGT: (**a**) Conventional SGT; (**b**) FPI-SGT, where the ellipsis indicates that the floating P-islands are repeated in the vertical direction.

Figure 1 shows the two-dimensional cross-sectional structures of the Conventional SGT and FPI-SGT. Both devices use the same basic shielded-gate trench cell, which mainly consists of a trench gate, source-connected shield polysilicon, gate oxide, inter-polysilicon oxide, a P-type body region, an N^+^ source region, an N-type epitaxial drift region, and an N^+^ substrate. Compared with the Conventional SGT, the FPI-SGT introduces discrete floating P-islands vertically into the N-type drift region beneath the source-connected shield polysilicon.

Each floating P-island is located beneath the source-connected shield polysilicon and arranged vertically in the device. None of the islands is directly connected to the source, gate, or drain, and its potential is determined self-consistently by electrostatic coupling with the surrounding drift region. In the off state, the local P/N junctions formed between the P-islands and the surrounding N-type drift region participate in depletion and introduce additional electric-field peaks inside the drift region, thereby improving the vertical electric-field distribution of the device.

To ensure consistency in the comparison among different structures, the cell pitch, trench dimensions, gate-oxide thickness, P-type body region, source-region structure, and substrate parameters are kept identical except for the number of floating P-islands. The specific values are listed in Table 1. The basic parameters of the Conventional SGT are taken from an established 100 V-class SGT MOSFET model that has been benchmarked against experimental data, whereas the floating-P-island parameters are determined from the device dimensions and preliminary parameter sweeps. The nominal doping concentration of each P-island is set to 1.2 × 10^17^ cm^−3^, and the N-type drift-region doping concentration *N_epi_* is used as the device-optimization variable.

Two-dimensional simulations of the device electrical characteristics are performed using Sentaurus TCAD (Version T-2022.03, Synopsys, Inc., Mountain View, CA, USA). The simulation temperature is set to 300 K, and doping-dependent mobility, normal-electric-field-dependent mobility, high-field velocity saturation, Shockley–Read–Hall (SRH) recombination, Auger recombination, and the Slotboom bandgap-narrowing model are adopted. The Lackner impact-ionization model is included in the high-voltage off-state simulations [23,24,25].

**Figure 2:**
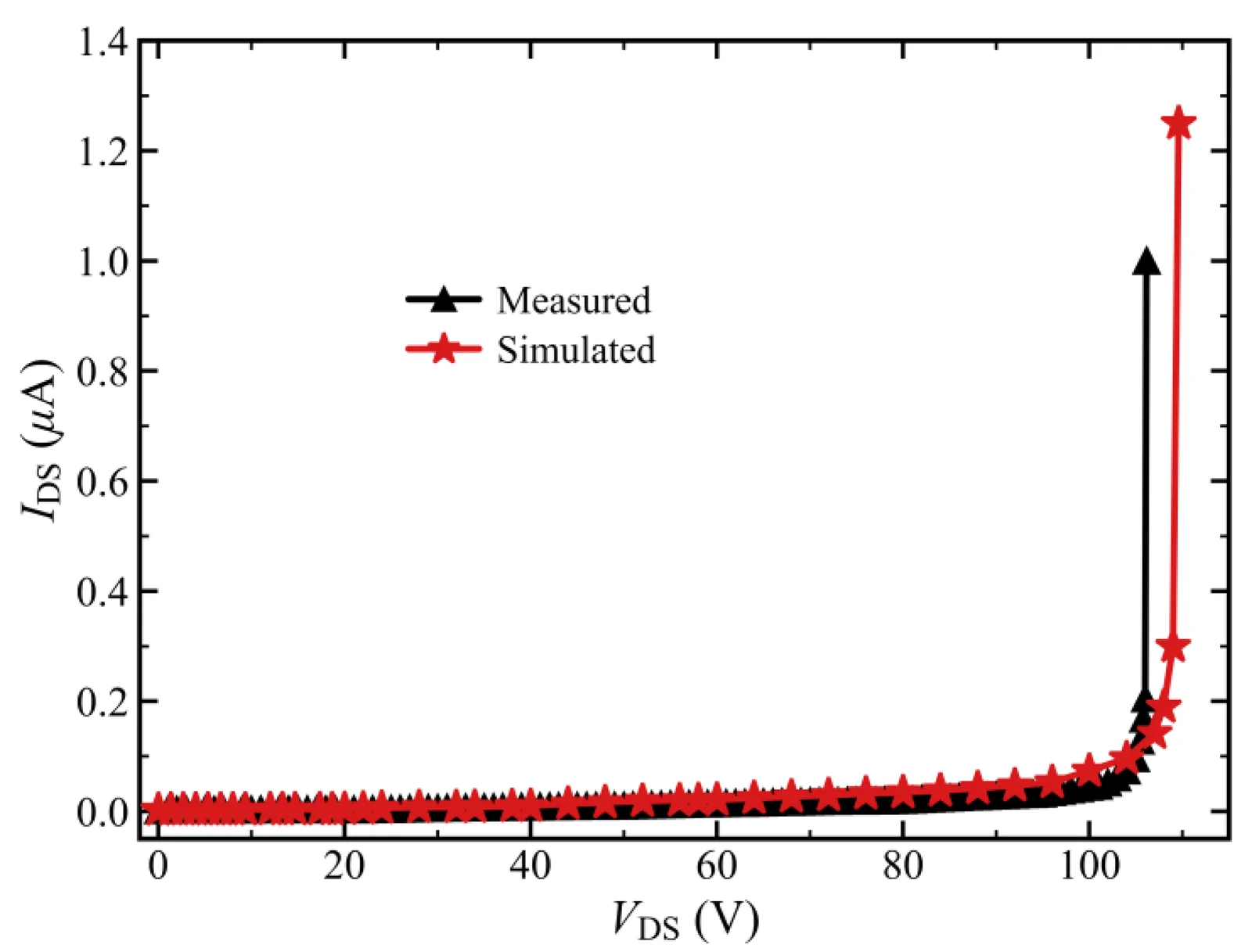
Comparison of measured and simulated off-state characteristics of a 100 V N-channel SGT MOSFET.

Using the above physical models, a 100 V-rated N-channel SGT MOSFET is simulated and compared with measured data. As shown in Figure 2, the simulated and measured curves agree well, indicating that the adopted TCAD model can reasonably reproduce the off-state characteristics of the SGT MOSFET.

The device breakdown voltage is extracted using a specified drain-current-density criterion: when the off-state drain-current density reaches 0.23 mA/cm^2^, the corresponding drain–source voltage is defined as BV, and all drain currents are normalized to the complete cell area as current densities. This criterion is consistent with that used to extract BV for the experimental device above and is applied throughout this work as a unified engineering criterion for comparing the off-state blocking capability of different structures at the same leakage-current level. *R_on,sp_* is extracted from the slope of the *I_D_*–*V_DS_* curve in the low-*V_DS_* linear region at *V_GS_* = 10 V, and FOM is defined as BV^2^/*R_on,sp_*. The same simulation and parameter-extraction conditions are applied to all structures.

## 3. Electric-Field Modulation in the off State

### 3.1. Off-State Performance Limitations of the Conventional SGT

**Figure 3:**
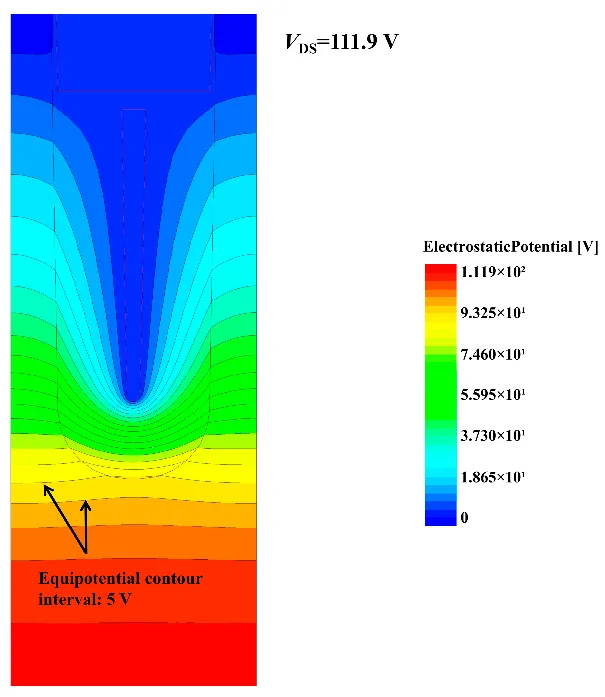
Off-state potential distribution of the Conventional SGT at 111.9 V (5 V/contour).

Under the above structural parameters and unified simulation conditions, Figure 3 shows the potential distribution of the Conventional SGT in the off state. When the N-type drift-region doping concentration is *N_epi_* = 8.0 × 10^15^ cm^−3^, the BV extracted according to the above leakage-current-density criterion is 111.9 V. The calculated ionization integral is far below 1, indicating that the device has not reached the self-sustaining avalanche condition. Therefore, the BV extracted in this study represents the off-state blocking capability at the specified leakage-current level, and the following analysis focuses mainly on the potential distribution to clarify the limitation of the off-state performance.

As shown in Figure 3, the equipotential lines are clearly dense near the trench bottom and gradually become sparse in the lower drift region, indicating that the potential gradient is mainly concentrated near the trench bottom and that the lower drift region is not fully utilized. Therefore, under the above structural parameters, the off-state performance of the Conventional SGT is mainly limited by local electric-field concentration at the trench bottom and insufficient utilization of the drift region for voltage blocking.

Increasing the BV of the Conventional SGT generally requires reducing the drift-region doping concentration or increasing the epitaxial-layer thickness; however, both approaches increase the drift-region resistance and hence *R_on,sp_*, thereby limiting further optimization of the BV–*R_on,sp_* trade-off. Therefore, an additional electric-field modulation structure must be introduced into the drift region to improve the vertical electric-field distribution and voltage-blocking utilization.

### 3.2. Effect of the Number of P-Islands

**Figure 4:**
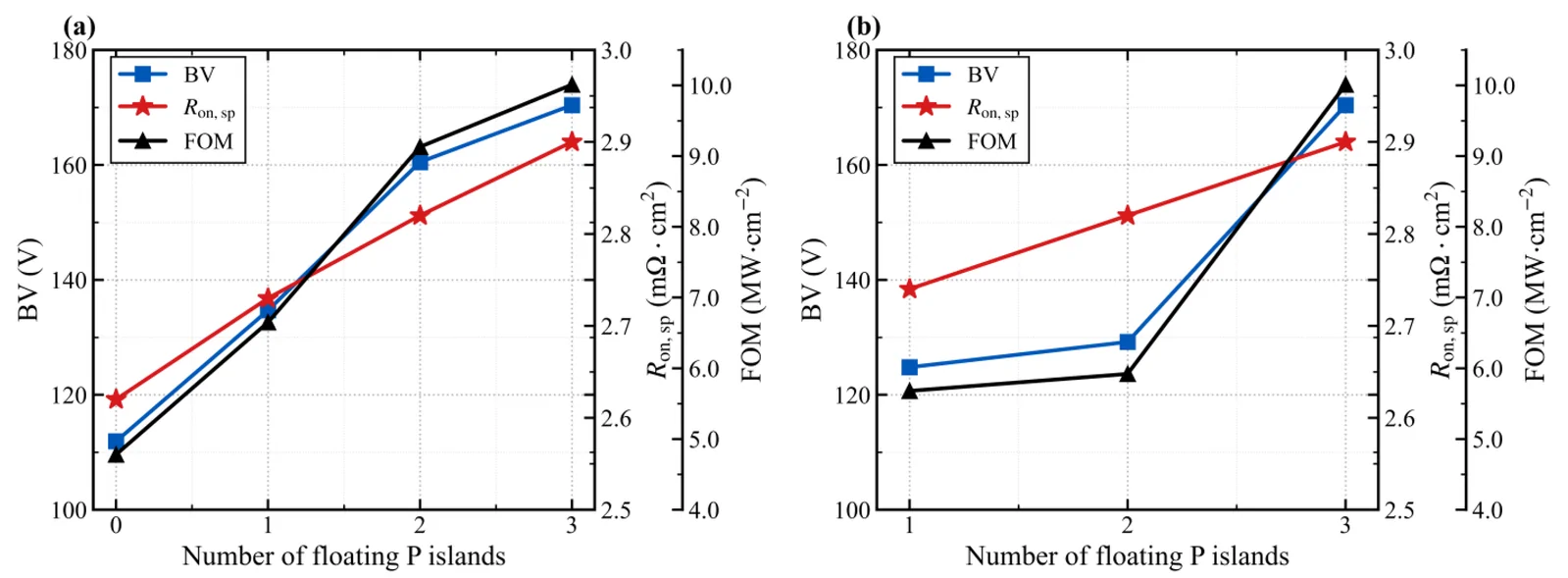
Variations in device BV, *R_on,sp_*, and FOM with the number of floating P-islands: (**a**) fixed size and doping concentration for each P-island; (**b**) fixed total P-type compensation charge.

To analyze the effect of the number of floating P-islands on the static performance of the device, while also considering that increasing the island number in practical fabrication would increase the complexity of repeated epitaxial growth, local ion implantation, and vertical alignment, the investigated range is limited to 0–3 floating P-islands. At a fixed *N_epi_* of 8.0 × 10^15^ cm^−3^, Figure 4a compares the BV, *R_on,sp_*, and FOM of the Conventional SGT and the structures containing one, two, and three floating P-islands. Figure 4b further compares the one-, two-, and three-island structures while keeping the total P-type compensation charge constant.

As shown in Figure 4a, when the number of floating P-islands increases from 0 to 3, the device BV continuously increases from 111.9 to 170.4 V, corresponding to an improvement of approximately 52.3%, indicating that the introduction of floating P-islands effectively enhances the off-state voltage-blocking capability of the drift region. Meanwhile, *R_on,sp_* increases from 2.62 mΩ·cm^2^ for the Conventional SGT to 2.90 mΩ·cm^2^ for the 3FPI-SGT. This increase occurs because the floating P-islands occupy part of the N-type conduction region and cause the on-state current path to detour, reducing the effective conductive cross-sectional area of the drift region. Although *R_on,sp_* increases with the number of floating P-islands, the increase in BV is more pronounced, causing FOM to increase from approximately 4.78 to 10.01 MW·cm^−2^.

Because the size and doping concentration of each P-island are fixed in Figure 4a, increasing the number of islands also increases the total P-type compensation charge. To decouple these two effects, Figure 4b uses the 3FPI-SGT as the reference, for which the doping concentration of each P-island is 1.2 × 10^17^ cm^−3^. With the size of each P-island kept unchanged, the doping concentration of each island in the two-island and one-island structures is set to 1.8 × 10^17^ and 3.6 × 10^17^ cm^−3^, respectively, so that the three structures have the same total P-type compensation charge. The results show that, even at an identical total compensation charge, the BV and FOM of the three-island structure remain significantly higher than those of the one- and two-island structures. This indicates that the performance improvement does not arise solely from the increase in the total P-type compensation charge; the number and spatial distribution of the P-islands also play important roles.

Within the range of 0–3 floating P-islands investigated in this study, the 3FPI-SGT achieves the highest BV and FOM and exhibits the best BV–*R_on,sp_* trade-off. Therefore, the 3FPI-SGT is selected for the subsequent analysis.

### 3.3. Multi-Peak Electric-Field Modulation Mechanism of the 3FPI-SGT

**Figure 5:**
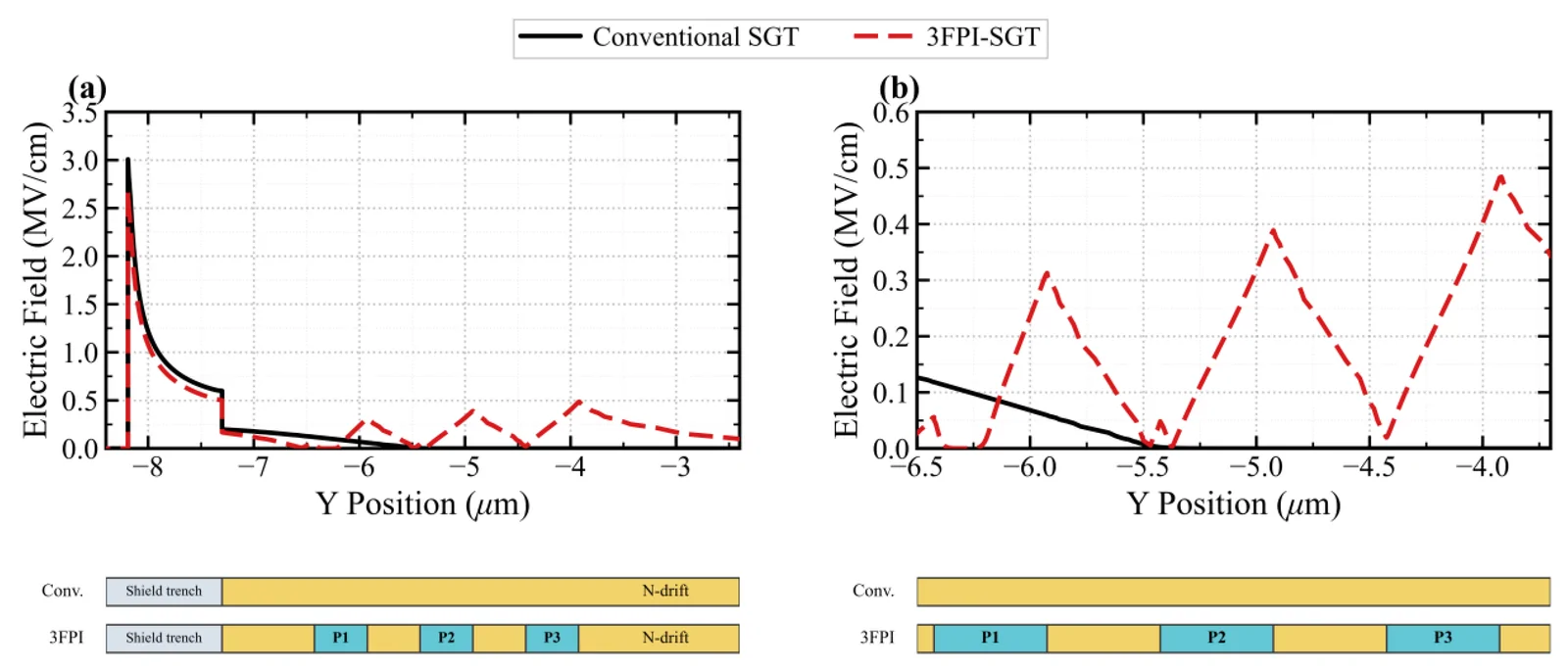
Vertical electric-field distributions of the Conventional SGT and the 3FPI-SGT under their corresponding high-voltage off-state conditions: (**a**) overall vertical electric-field distribution; (**b**) enlarged view of the region containing the floating P-islands. The color blocks beneath the plots indicate the corresponding internal structures along the same vertical coordinate.

To further clarify the physical mechanism by which the 3FPI-SGT increases BV and improves the BV–*R_on,sp_* trade-off, Figure 5 compares the vertical electric-field distributions of the Conventional SGT and the 3FPI-SGT in the off state at their respective criterion-based BVs. The electric-field profiles are extracted vertically from the trench bottom toward the drift region and cover the region containing the three floating P-islands.

As shown in Figure 5a, the electric field of the Conventional SGT is mainly concentrated near the trench bottom, where the main electric-field peak is approximately 3.01 MV/cm, and then rapidly decays vertically along the drift region. This indicates that the potential drop is mainly concentrated near the trench bottom, whereas the voltage-blocking capability of the lower drift region is not fully utilized. In contrast, the main electric-field peak at the trench bottom of the 3FPI-SGT is reduced to approximately 2.68 MV/cm, which is approximately 10.9% lower than that of the Conventional SGT. Meanwhile, multiple local electric-field peaks are formed inside the drift region of the 3FPI-SGT, so its vertical electric field no longer exhibits a monotonic decay from the trench bottom toward the drift region.

Figure 5b further enlarges the electric-field distribution in the region containing the floating P-islands. In the Conventional SGT, the electric field in this region gradually decreases with vertical position, without an evident local field-strength peak. In contrast, the 3FPI-SGT exhibits pronounced electric-field fluctuations near the floating P-islands, with three prominent secondary peaks of approximately 0.31, 0.39, and 0.48 MV/cm. As these peaks extend from the trench bottom into the drift region, their values show an overall increasing trend, indicating that the lower drift region progressively participates in supporting the applied voltage.

These results indicate that the local P/N junctions formed between the floating P-islands and the surrounding N-type drift region alter the depletion state and potential gradient in the drift region, redistributing part of the potential drop originally concentrated near the trench bottom into the drift region. Consequently, the 3FPI-SGT develops a vertical electric-field distribution consisting of the main peak at the trench bottom and multiple secondary peaks inside the drift region. This distribution not only reduces local electric-field concentration at the trench bottom but also improves the overall utilization of the drift region for voltage blocking. This is an important physical reason for the higher BV and FOM of the 3FPI-SGT.

## 4. Drift-Region Doping Optimization and Static/Dynamic Performance Evaluation

Section 3 shows that floating P-islands can improve the vertical electric-field distribution of the drift region through multi-peak electric-field modulation. This modulation is affected by the matching relationship between the N-type drift-region doping concentration *N_epi_* and the P-island parameters. This section first investigates the effect of *N_epi_* on the static performance of the Conventional SGT and the 3FPI-SGT and selects operating points with identical *R_on,sp_* values for a fair comparison. The parasitic capacitances, gate charge, and double-pulse switching characteristics of the two structures are then analyzed at the matched operating points to evaluate the dynamic-performance penalty accompanying the static-performance improvement.

### 4.1. Static Performance: Drift-Region Doping Optimization and R_on,sp_ Matching

**Figure 6:**
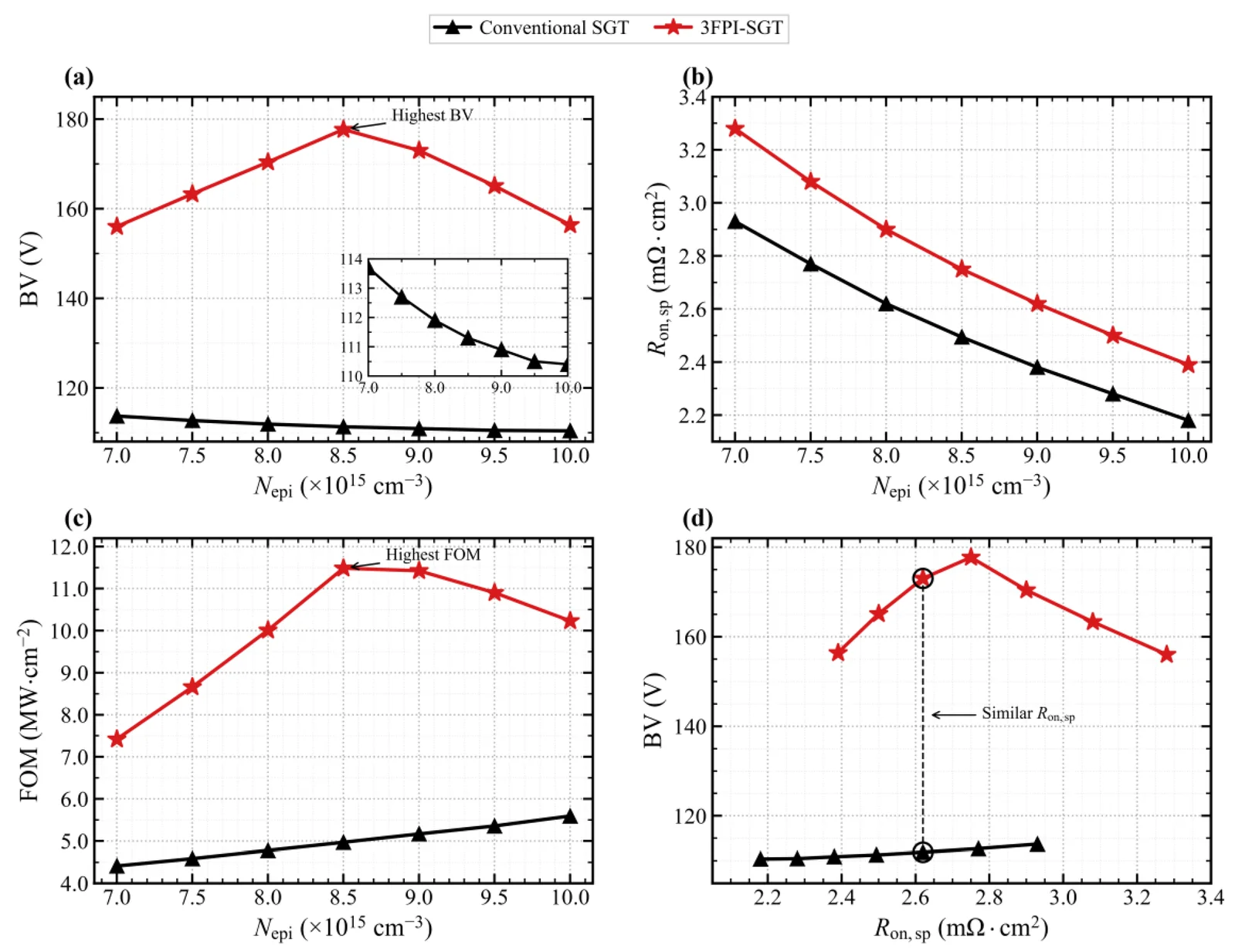
Comparison of the static performance of the Conventional SGT and the 3FPI-SGT at different *N_epi_* values: (**a**) BV–*N_epi_*; (**b**) *R_on,sp_*–*N_epi_*; (**c**) FOM–*N_epi_*; (**d**) BV–*R_on,sp_* trade-off.

Figure 6 shows the BV, *R_on,sp_*, FOM, and BV–*R_on,sp_* trade-off of the Conventional SGT and the 3FPI-SGT over the *N_epi_* range of 7.0 × 10^15^–1.0 × 10^16^ cm^−3^. As *N_epi_* increases, *R_on,sp_* decreases monotonically for both structures: from 2.93 to 2.18 mΩ·cm^2^ for the Conventional SGT and from 3.28 to 2.39 mΩ·cm^2^ for the 3FPI-SGT. Because the floating P-islands occupy part of the N-type conduction region and cause the current path to detour, the *R_on,sp_* of the 3FPI-SGT is slightly higher than that of the Conventional SGT at the same *N_epi_*.

The BV variations of the two structures with *N_epi_* are markedly different. The BV of the Conventional SGT decreases only slowly from 113.7 to 110.4 V, indicating that its blocking capability is mainly limited by local electric-field concentration at the trench bottom and is insensitive to changes in drift-region doping. In contrast, the BV of the 3FPI-SGT first increases and then decreases, reaching 177.7 V at *N_epi_* = 8.5 × 10^15^ cm^−3^. When *N_epi_* is too low or too high, the matching between the drift-region charge and the depletion charge of the P-islands deteriorates, causing the vertical electric-field distribution in the drift region to deviate from the preferable state; at a moderate *N_epi_*, the charge matching is more appropriate, resulting in a higher BV. The FOM exhibits the same trend, reaching 11.48 MW·cm^−2^ for the 3FPI-SGT at *N_epi_* = 8.5 × 10^15^ cm^−3^, which is significantly higher than the 4.41–5.59 MW·cm^−2^ of the Conventional SGT over this doping range.

To compare the blocking performance at the same conduction loss and quantify the gain provided by the floating-P-island structure, Figure 6d selects two operating points with the same *R_on,sp_* of 2.62 mΩ·cm^2^: *N_epi_* = 8.0 × 10^15^ cm^−3^ and BV = 111.9 V for the Conventional SGT, and *N_epi_* = 9.0 × 10^15^ cm^−3^ and BV = 173.0 V for the 3FPI-SGT. At identical *R_on,sp_*, the BV of the 3FPI-SGT is increased by approximately 54.6%, and the FOM increases from 4.78 to 11.42 MW·cm^−2^. The subsequent comparisons of dynamic characteristics use these two matched operating points.

### 4.2. Dynamic Characteristics: Parasitic Capacitances, Gate Charge, and Switching Losses

**Figure 7:**
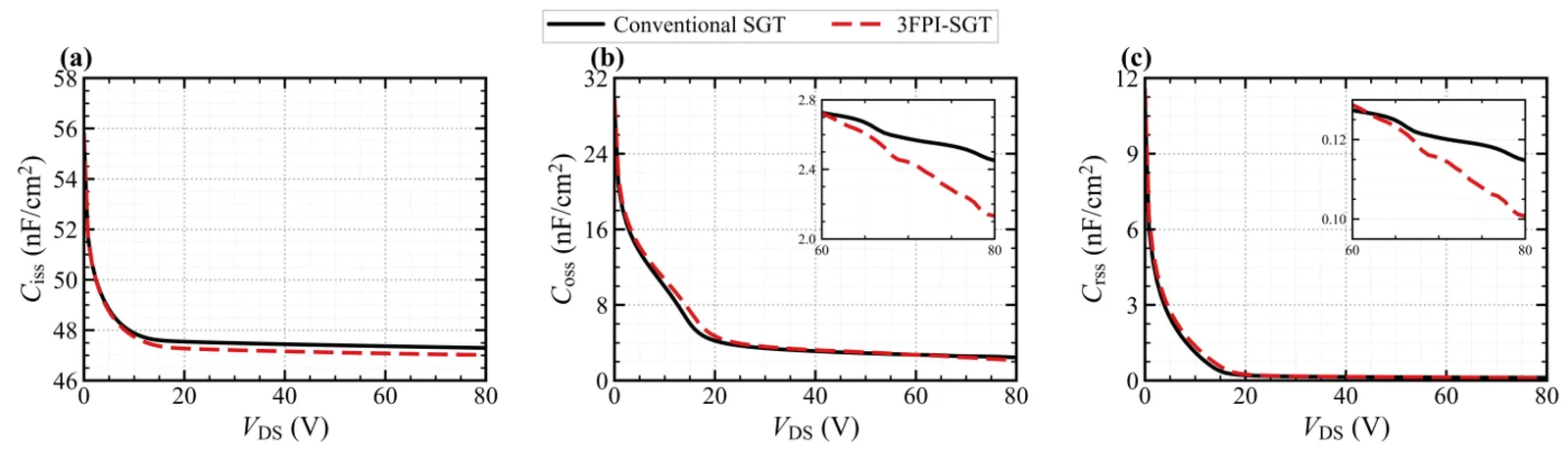
Comparison of the parasitic capacitances of the Conventional SGT and 3FPI-SGT: (**a**) *C_iss_*–*V_DS_*; (**b**) *C_oss_*–*V_DS_*; (**c**) *C_rss_*–*V_DS_* (the insets enlarge the *V_DS_* = 40–80 V range).

Parasitic-capacitance simulations are performed using Sentaurus MixedMode small-signal AC analysis. The source and gate are held at 0 V, the drain voltage is swept from 0 to 80 V, and a 1 MHz small-signal AC analysis is carried out at each DC bias point. *C_iss_*, *C_oss_*, and *C_rss_* are extracted from the three-terminal small-signal capacitance matrix. Figure 7 compares the capacitances of the two structures under *R_on,sp_*-matched conditions. At *V_DS_* < 20 V, the corresponding capacitance curves are generally close, indicating that the floating P-islands have little effect on the terminal capacitances in this range. The *C_iss_* curves of the two devices remain very close over the entire drain-voltage range, with a relative difference of less than 1% in the high-voltage region, indicating that the floating-P-island structure and the drift-region doping adjustment have only a limited effect on the input capacitance. This is because *C_iss_* is mainly determined by the gate–source capacitance associated with the trench-gate structure, whereas the P-islands are located inside the drift region, far from the gate region. As *V_DS_* increases further, *C_oss_* and *C_rss_* exhibit different voltage dependences for the two devices and gradually separate above approximately 60 V. At *V_DS_* = 80 V, *C_oss_* and *C_rss_* of the Conventional SGT are 2.45 nF/cm^2^ and 0.115 nF/cm^2^, respectively, whereas those of the 3FPI-SGT are 2.14 nF/cm^2^ and 0.101 nF/cm^2^, corresponding to reductions of approximately 12.8% and 12.5%. This result indicates that, at high *V_DS_*, the expanded depletion regions around the floating P-islands weaken charge coupling between the drain and the gate/source, thereby reducing the drain-related capacitances.

**Figure 8:**
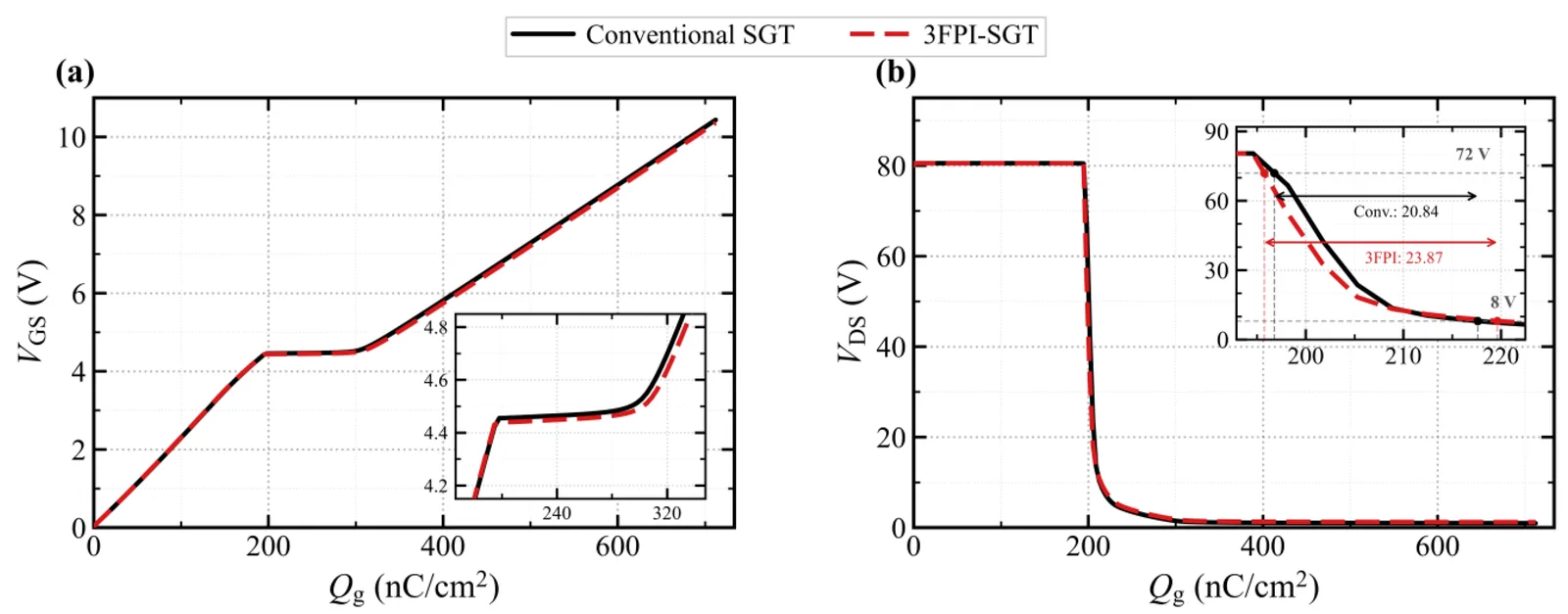
Comparison of gate charge for the Conventional SGT and 3FPI-SGT at *V_DS_* = 80 V: (**a**) *V_GS_*–*Q_g_*; (**b**) *V_DS_*–*Q_g_*.

Gate-charge simulations are performed using a Sentaurus MixedMode constant-current gate-charging circuit. The DC bus voltage is set to 80 V. After the drain operating state is established, the gate is charged by a constant-current source, and *Q_g_* is obtained by time integration of the gate current. *Q_gd_* is defined as the change in gate charge during the interval in which *V_DS_* decreases from 90% to 10% of the test voltage (i.e., from 72 to 8 V). Figure 8 shows the gate-charge characteristics of the two structures, and Table 2 lists the total gate charge *Q_g_*, Miller charge *Q_gd_*, and Miller plateau voltage. The *Q_g_* values of the Conventional SGT and the 3FPI-SGT are 682.9 and 687.9 nC/cm^2^, respectively. Their Miller plateau voltages are 4.456 and 4.441 V, respectively, a relative difference of only approximately 0.34%, which is consistent with their similar input-capacitance characteristics.

Compared with the Conventional SGT, *Q_gd_* of the 3FPI-SGT increases from 20.84 to 23.87 nC/cm^2^, an increase of approximately 14.5%. This is not inconsistent with its lower *C_rss_* in the high-*V_DS_* range of approximately 60–80 V. *C_rss_* represents the small-signal differential coupling at a fixed bias, whereas *Q_gd_* is the large-signal integrated gate charge over the entire Miller interval from 72 to 8 V. At high *V_DS_*, expansion of the depletion regions around the floating P-islands enhances shielding of the drain-side electric field and reduces the effective gate–drain coupling. As *V_DS_* decreases, the effective reverse bias of the P/N junctions around the P-islands weakens, the depletion regions contract, and the junction-depletion capacitance increases, thereby strengthening the incremental charge coupling between the floating P-islands and the surrounding drift region. This behavior is consistent with the increase in *C_gd,eff_* in the low-*V_DS_* range. Consequently, the additional integrated gate–drain charge contributed at low *V_DS_* exceeds the reduction at high *V_DS_*, leading to an overall increase in *Q_gd_*. Numerical differentiation, segmented integration, and voltage-step verification are provided in Supplementary Figure S1 and Table S1. Because the two matched operating points have the same *R_on,sp_*, *R_on,sp_* × *Q_gd_* increases by the same approximately 14.5%, making it the only switching figure of merit considered here that shows a noticeable deterioration.

**Figure 9:**
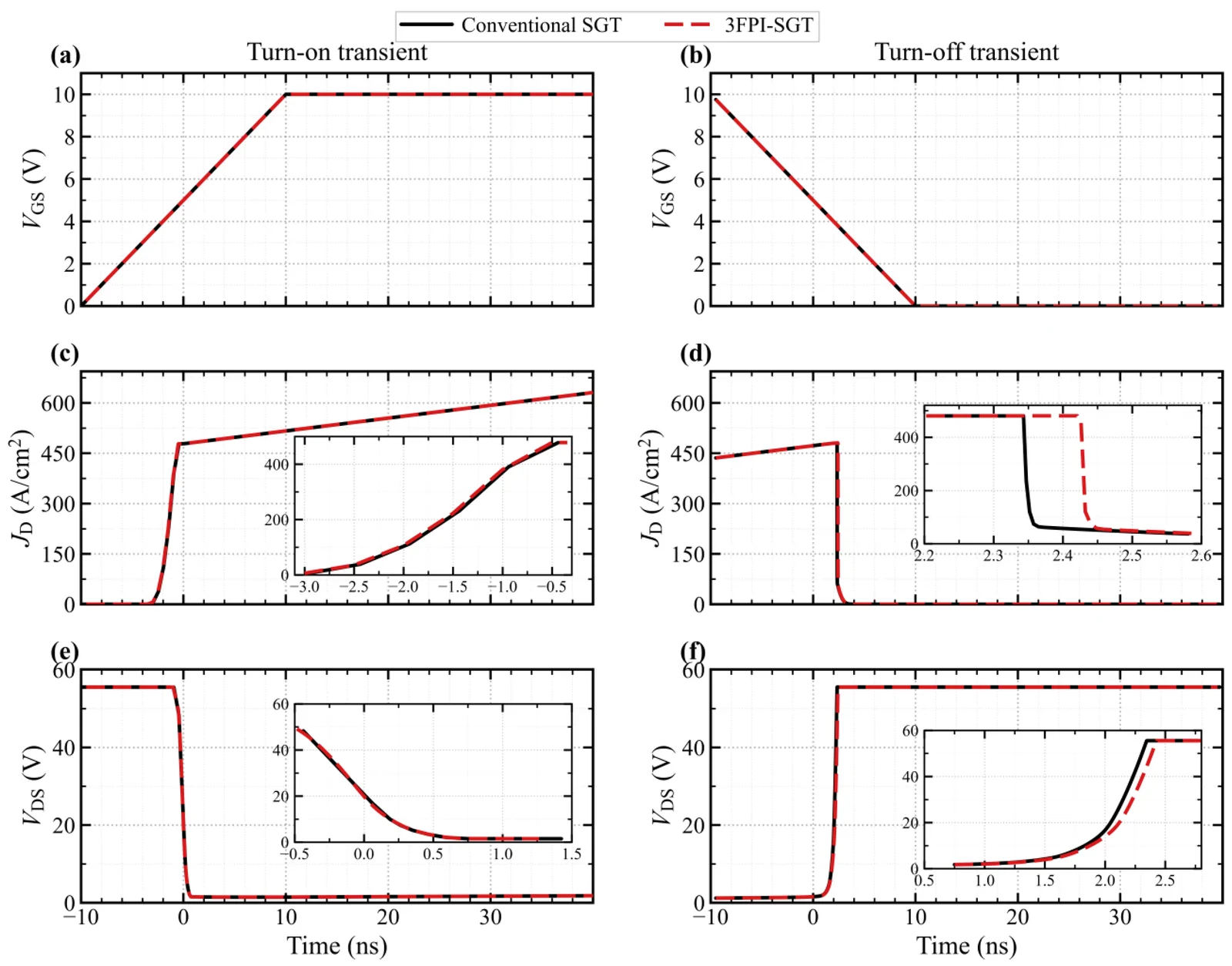
Double-pulse switching transient waveforms of the Conventional SGT and 3FPI-SGT: (**a**,**c**,**e**) *V_GS_*, *J_D_*, and *V_DS_* during turn-on; (**b**,**d**,**f**) *V_GS_*, *J_D_*, and *V_DS_* during turn-off.

**Figure 10:**
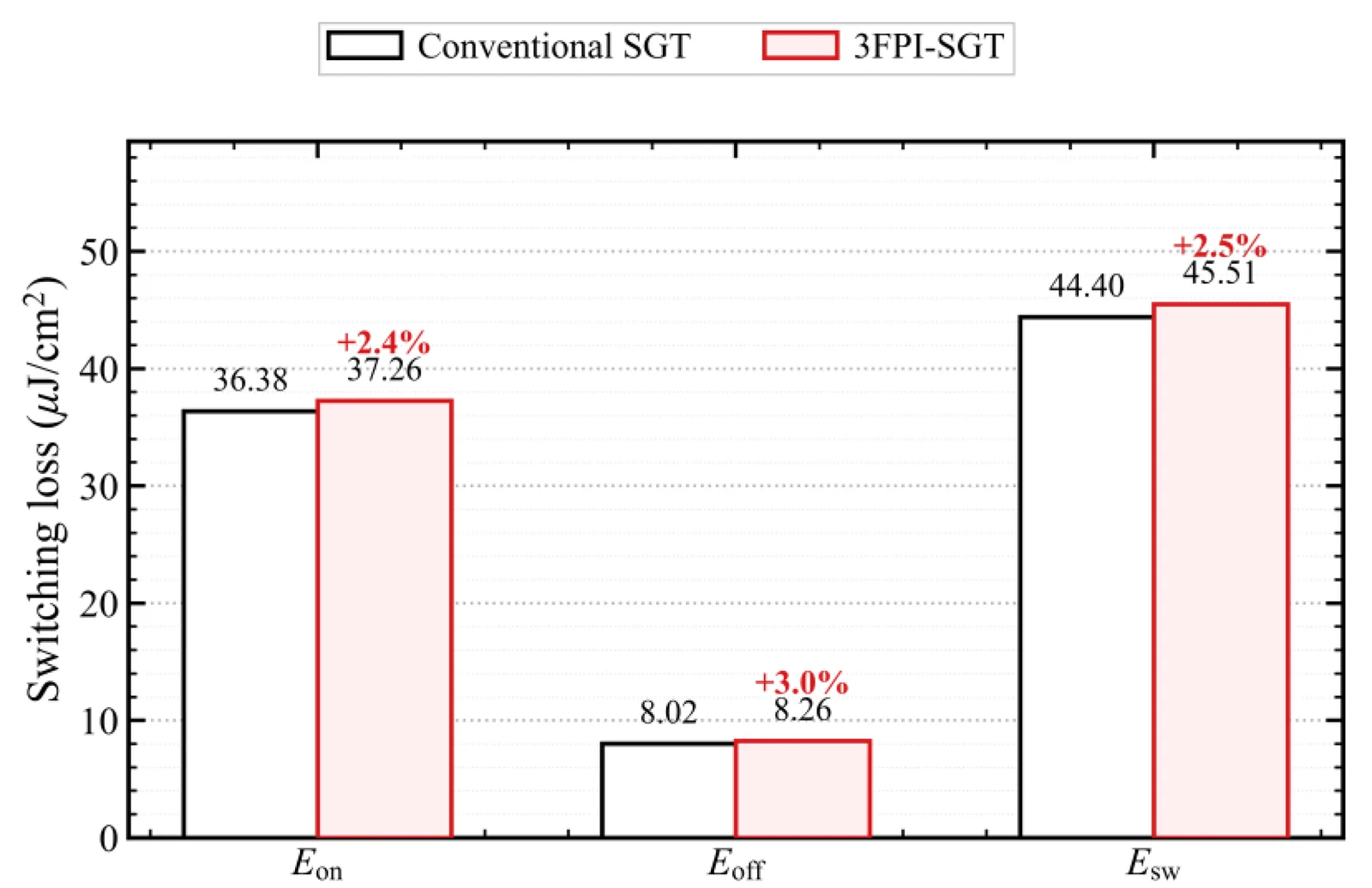
Comparison of switching energies (*E_on_*, *E_off_*, and *E_sw_*) between the Conventional SGT and 3FPI-SGT.

Double-pulse simulations are performed using a Sentaurus MixedMode inductive-load circuit consisting of a DC bus, load inductor, freewheeling diode, device under test, and gate resistor. The conditions are *V_DD_* = 55 V, *V_GS_* = 0–10 V, *R_g_* = 10 Ω, and *L_load_* = 0.5 H, and the same external circuit and gate-drive conditions are used for both devices. *E_on_* and *E_off_* are obtained by integrating *V_DS_*·*J_D_* over the turn-on and turn-off transition intervals, respectively. Figure 9 shows the double-pulse switching transient waveforms of the two structures. The *V_GS_*, *J_D_*, and *V_DS_* curves of the Conventional SGT and 3FPI-SGT nearly overlap during both turn-on and turn-off, indicating that the floating P-islands do not significantly change the switching transition speed. Using the same switching-transition integration criterion, Figure 10 further compares the switching energies: *E_on_* increases from 36.38 to 37.26 µJ/cm^2^ (+2.4%), *E_off_* from 8.02 to 8.26 µJ/cm^2^ (+3.0%), and the total switching energy *E_sw_* from 44.40 to 45.51 µJ/cm^2^ (+2.5%). The slight increase in switching loss is consistent with the increase in *Q_gd_*, but its magnitude is much smaller than the improvements in static BV and FOM, indicating that the overall dynamic-performance penalty of the 3FPI-SGT is limited.

In addition, because the drift-region doping concentrations of the Conventional SGT and the 3FPI-SGT are different under *R_on,sp_*-matched conditions, Table 3 summarizes the changes in dynamic characteristics among four operating points to distinguish the effects of the floating-P-island structure from those of the drift-region doping adjustment.

At both *N_epi_* = 8 × 10^15^ and 9 × 10^15^ cm^−3^, introducing floating P-islands reduces both *C_oss_* and *C_rss_*, while *Q_gd_* and *E_sw_* increase only slightly, consistent with the overall trend observed under *R_on,sp_*-matched conditions. This shows that, even after eliminating the difference in drift-region doping, the floating-P-island structure itself produces clear changes in the dynamic characteristics. Therefore, the dynamic differences observed at the *R_on,sp_*-matched operating points cannot be attributed simply to drift-region doping adjustment; rather, they result from the combined effects of the floating-P-island structure and the doping change.

Overall, under *R_on,sp_*-matched conditions, the principal advantage of the 3FPI-SGT is the approximately 54.6% increase in BV. Its *C_iss_* and total gate charge *Q_g_* remain essentially unchanged; at *V_DS_* = 80 V, *C_oss_* and *C_rss_* decrease by approximately 12.8% and 12.5%, respectively. Meanwhile, *Q_gd_* increases by approximately 14.5%, whereas the total switching energy increases by only approximately 2.5%. Thus, the static-performance gain introduced by the floating P-islands is substantially larger than the associated dynamic-performance penalty.

## 5. Process Robustness and Implementation Considerations

### 5.1. Sensitivity to Parameter Deviations

Using the 3FPI-SGT at the *R_on,sp_*-matched operating point in Section 4.1 (*N_epi_* = 9.0 × 10^15^ cm^−3^, BV = 173.0 V, *R_on,sp_* = 2.62 mΩ·cm^2^) as the baseline and keeping all other parameters unchanged, the BV variation is examined under single-factor deviations in the P-island doping concentration *N_PI_*, island spacing *G_PI_*, and the vertical spacing *S_PI_* between the bottom oxide of the shield-gate trench and the upper edge of the topmost floating P-island. When *N_PI_* deviates by ±10%, BV decreases to 164.74–167.7 V; when the deviation expands to ±20%, BV further decreases to 153.8–157.9 V. BV is lower than the nominal value for both increased and decreased *N_PI_*, indicating the existence of a preferable matching range between the P-island doping and the drift-region charge, with the nominal *N_PI_* already close to that range. By comparison, ±10% or ±20% deviations in *G_PI_* change BV only slightly, to approximately 170–171 V. When *S_PI_* varies by ±10% or ±20% from its nominal value, BV remains within 163.6–171.3 V, with a maximum reduction of approximately 5.4%, indicating some tolerance to this vertical-spacing deviation.

Overall, the sensitivity of BV to the three parameter deviations decreases in the order *N_PI_*, *S_PI_*, and *G_PI_*. Even under the most unfavorable ±20% *N_PI_* deviation, the BV of the 3FPI-SGT remains no lower than 153.8 V, approximately 37.4% higher than the Conventional SGT value of 111.9 V, while *R_on,sp_* remains approximately 2.62 mΩ·cm^2^. This indicates that the core static advantage of the proposed structure is maintained within the investigated range of single-factor process deviations.

### 5.2. Conceptual Fabrication Process

**Figure 11:**
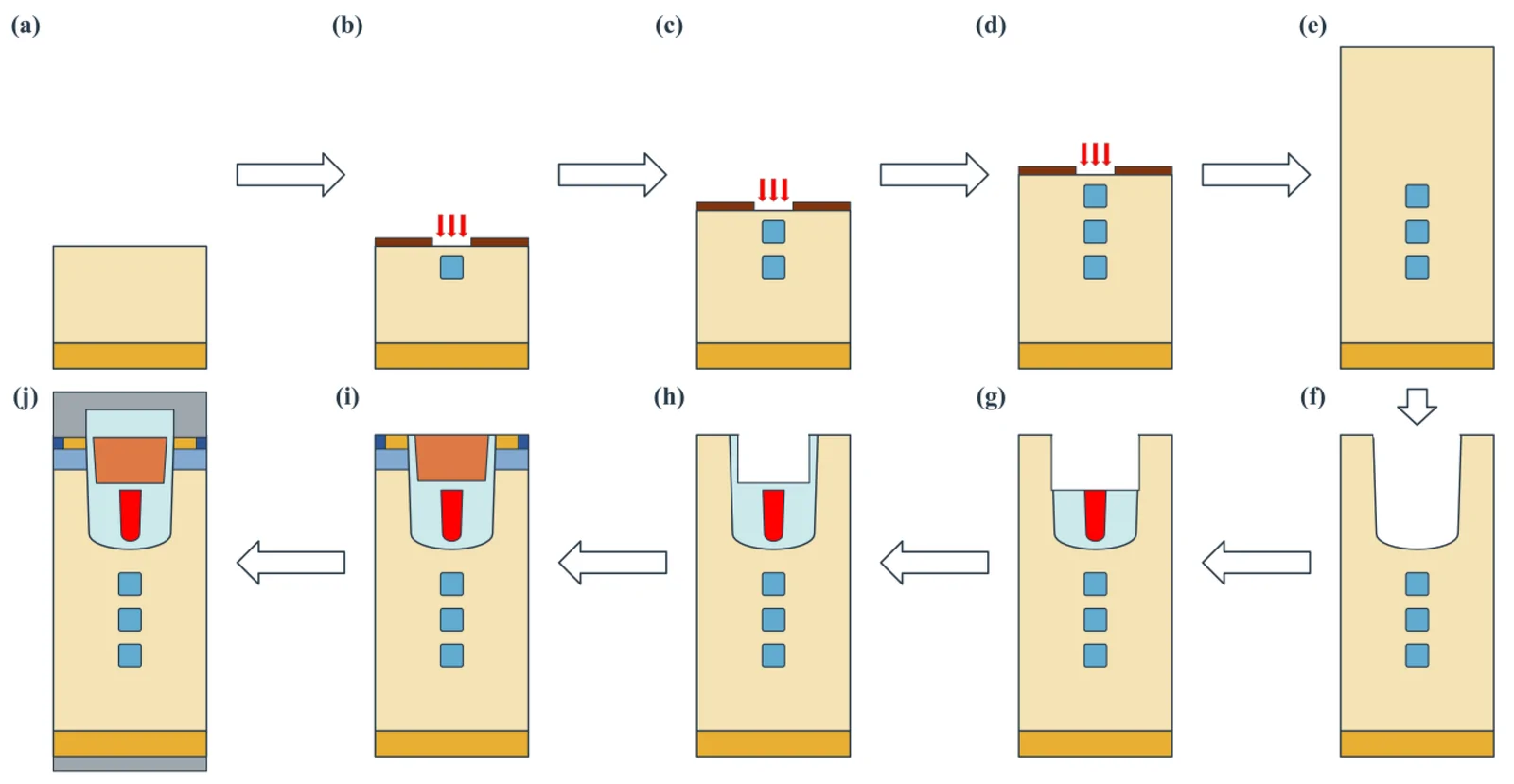
Key fabrication process flow for the 3FPI-SGT MOSFET: (**a**) growth of the first N^−^ epitaxial layer; (**b**) formation of the bottom floating P-island; (**c**) growth of the second N^−^ epitaxial layer and formation of the middle floating P-island; (**d**) growth of the third N^−^ epitaxial layer and formation of the upper floating P-island; (**e**) final N^−^ epitaxial overgrowth and dopant activation; (**f**) deep-trench etching; (**g**) formation of the shield oxide and shield polysilicon; (**h**) formation of the inter-polysilicon dielectric and gate oxide; (**i**) formation of the gate polysilicon, P-type body, N^+^ source, and P^+^ body-contact regions; (**j**) contact-hole formation and front-side/backside metallization.

To assess the fabrication feasibility of the three-floating-P-island-assisted shielded-gate trench MOSFET (3FPI-SGT), Figure 11 presents a conceptual process flow. The required unit processes—stepwise epitaxial growth, masked boron ion implantation, deep-trench etching, oxide growth, and polysilicon filling—can all be implemented using existing silicon power-device process modules, demonstrating feasibility at the unit-process level. First, N^−^ epitaxial layers are grown stepwise on an N^+^ substrate, and the lower, middle, and upper floating P-islands are sequentially formed by masked boron implantation. A final epitaxial cap layer is then grown and the dopants are activated. A deep trench is subsequently etched, followed by formation of the shield oxide, shield polysilicon, inter-polysilicon dielectric, gate oxide, and gate polysilicon. The P-type body, N^+^ source, and P^+^ body-contact regions are then formed, and the process is completed by contact-hole formation and front-side source and backside drain metallization.

This scheme uses discrete floating P-islands to modulate the drift-region electric field and avoids the direct formation of a continuous deep P-pillar; however, it increases the number of epitaxial-growth and local-implantation steps and imposes tighter control requirements on epitaxial thickness, implantation dose, interlayer alignment, and thermal budget. Subsequent high-temperature processing may also cause vertical and lateral boron diffusion, altering the size, spacing, and doping distribution of the P-islands. Therefore, the manufacturability assessment in this work is based mainly on compatibility with existing process modules and the parameter-deviation analysis in Section 5.1. The specific process window, alignment tolerance, yield, and manufacturing cost still require further validation on an actual process platform and through sample fabrication.

## 6. Conclusions

To address trench-bottom electric-field concentration and insufficient utilization of the drift region for voltage blocking in the Conventional SGT, this work proposes a silicon shielded-gate trench MOSFET containing multiple vertically discrete floating P-islands and systematically investigates the performance evolution as the number of P-islands increases from 0 to 3 using Sentaurus TCAD. In the off state, the local P/N junctions around the floating P-islands generate secondary electric-field peaks, allowing the main trench-bottom peak and the secondary peaks inside the drift region to jointly support the potential drop. At *N_epi_* = 8.0 × 10^15^ cm^−3^, the BV of the 3FPI-SGT increases from 111.9 V for the Conventional SGT to 170.4 V, an improvement of approximately 52.3%, while the FOM increases from 4.78 to 10.01 MW·cm^−2^, an improvement of approximately 109.5%.

Drift-region doping optimization shows that the 3FPI-SGT reaches a maximum BV of 177.7 V at *N_epi_* = 8.5 × 10^15^ cm^−3^. Under matched conditions with an identical *R_on,sp_* of 2.62 mΩ·cm^2^, its BV is approximately 54.6% higher than that of the Conventional SGT. In terms of dynamic characteristics, *C_iss_* and the total gate charge *Q_g_* of the 3FPI-SGT remain essentially unchanged; at *V_DS_* = 80 V, *C_oss_* and *C_rss_* decrease by approximately 12.8% and 12.5%, respectively. *Q_gd_* increases by approximately 14.5%, whereas the total switching energy increases by only approximately 2.5%. The comparison at identical *N_epi_* further confirms that the floating-P-island structure itself makes a clear contribution to these dynamic changes, while the differences at the matched operating points result from the combined effects of the floating-P-island structure and the drift-region doping adjustment.

Parameter-sensitivity analysis shows that the 3FPI-SGT retains a clear BV advantage when *N_PI_*, *G_PI_*, or *S_PI_* undergoes a ±20% single-factor deviation, indicating a degree of performance stability within the investigated parameter-deviation range. In addition, a conceptual fabrication process based on multiple N^−^ epitaxial-growth steps, local boron ion implantation, and a conventional SGT trench process is proposed, providing a reference for subsequent process-feasibility evaluation and experimental verification. Overall, multiple vertically discrete floating P-islands significantly improve the BV–*R_on,sp_* trade-off of silicon SGT MOSFETs through multi-peak electric-field modulation while introducing only a limited dynamic-performance penalty.

## Figures and Tables

**Table 1:** Main structural parameters of the devices.

Parameter	Symbol	Value	Unit
Cell pitch	*C_P_*	2.8	µm
N-type epitaxial-layer thickness	*T_epi_*	13.0	µm
Trench width	*W_t_*	1.9	µm
Trench depth	*T_t_*	5.7	µm
Gate-polysilicon depth	*T_g_*	1.3	µm
Shield-gate polysilicon length	*T_ts_*	3.31	µm
Gate-oxide thickness	*T_oxg_*	0.06	µm
Spacing from the bottom oxide of the shield-gate trench to the upper edge of the first P-island	*S_PI_*	0.88	µm
P-island width	*W_PI_*	0.5	µm
P-island height	*T_PI_*	0.5	µm
P-island spacing	*G_PI_*	0.5	µm
P-island doping concentration	*N_PI_*	1.2 × 10^17^	cm^−3^

Note: The parameters in the table correspond to the labels in the structural schematic.

**Table 2:** Comparison of gate charge and Miller plateau voltage for the two structures.

Structure	*Q_g_* (nC/cm^2^)	*Q_gd_* (nC/cm^2^)	Miller Plateau Voltage (V)
Conventional SGT	682.9	20.84	4.456
3FPI-SGT	687.9	23.87	4.441

**Table 3:** Summary of changes in dynamic characteristics under four comparison conditions.

Comparison Condition	Change in *C_oss_*	Change in *C_rss_*	Change in *Q_gd_*	Change in *E_sw_*
3FPI-SGT, 8 × 10^15^ cm^−3^ vs. Conventional SGT, 8 × 10^15^ cm^−3^	−31.0%	−31.5%	+7.3%	+1.22%
3FPI-SGT, 9 × 10^15^ cm^−3^ vs. Conventional SGT, 9 × 10^15^ cm^−3^	−16.9%	−17.0%	+2.4%	+1.04%
3FPI-SGT, 9 × 10^15^ cm^−3^ vs. 3FPI-SGT, 8 × 10^15^ cm^−3^	+26.4%	+27.8%	+6.7%	+1.27%
3FPI-SGT, 9 × 10^15^ cm^−3^ vs. Conventional SGT, 8 × 10^15^ cm^−3^	−12.8%	−12.5%	+14.5%	+2.50%

Note: *C_oss_* and *C_rss_* are evaluated at *V_DS_* = 80 V; *E_sw_* uses the same switching-transition integration criterion. Each percentage change is calculated relative to the reference object listed after “vs.” in the corresponding comparison.

## Data Availability

The data presented in this study are available within the article and its Supplementary Materials.

## Supplementary Materials

The following supporting information can be downloaded at: https://www.mdpi.com/article/10.3390/mi17090999/s1. Figure S1: Analysis of the origin of gate–drain charge in the 3FPI-SGT: (a) large-signal effective gate–drain capacitance *C_gd,eff_* = |d*Q_g_*/d*V_DS_*| obtained from the *Q_g_*(*V_DS_*) transient data using central differences; (b) accumulated charge Δ*Q_g_* of *C_gd,eff_* over *V_DS_* = 72–8 V, whose endpoint corresponds to *Q_gd_* in Figure 8 and Table 2 of the main text; Table S1: Comparison of gate–drain charge at different voltage-grid resolutions.
