# Supplementary material for: BV–Ron,sp Trade-Off Optimization in a Floating-P-Island-Assisted Silicon Shielded-Gate Trench MOSFET
Source: Micromachines (Basel). 2026 Aug 24;17(9):999. doi: 10.3390/mi17090999 (PMC13609020; doi:10.3390/mi17090999)
Supplement: Supplementary file 1 [file micromachines-17-00999-s001.zip › micromachines-4475238-supplementary.pdf]

Supplementary Materials

To explain the apparent difference between the reduction in C_{rss} in the high-V_{DS} range and the increase in Q_{gd} in the main text, the large-signal effective gate–drain capacitance $C_{gd,eff} = |dQ_g/dV_{DS}|$ is calculated from the $Q_g(V_{DS})$ transient data and integrated over the Miller interval in which V_{DS} decreases from 90% (72 V) to 10% (8 V) of the test voltage. Figure S1a shows the variation in $C_{gd,eff}$ with V_{DS}, and Figure S1b shows the corresponding accumulated charge. The results indicate that the $C_{gd,eff}$ of the 3FPI-SGT is lower in the higher-V_{DS} range but increases considerably in the lower-V_{DS} range. The integrated contribution of the latter exceeds the reduction in the high-voltage range; consequently, Q_{gd} ultimately increases from 20.84 nC/cm² for the Conventional SGT to 23.87 nC/cm² for the 3FPI-SGT.

To exclude the effects of numerical differentiation and interpolation step size on the results, the $Q_g(V_{DS})$ data are further reinterpolated at four voltage-grid resolutions of 0.5, 0.2, 0.1, and 0.05 V, and $C_{gd,eff}$ is calculated using central differences. Table S1 shows that, over the above grid range, the Q_{gd} values extracted for the two structures remain 20.84 and 23.87 nC/cm², respectively, indicating that the difference exhibits good grid independence under the present simulation conditions and is not caused by numerical-processing errors.

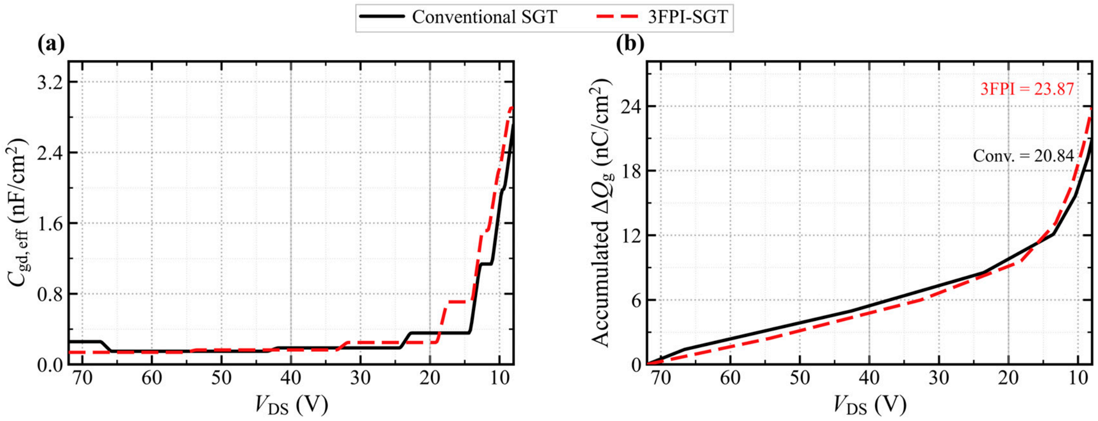

Figure S1. Analysis of the origin of gate–drain charge in the 3FPI-SGT: (**a**) large-signal effective gate–drain capacitance $C_{gd,eff} = |dQ_g/dV_{DS}|$ obtained from the $Q_g(V_{DS})$ transient data using central differences; (**b**) accumulated charge ΔQ_g of $C_{gd,eff}$ over V_{DS} = 72–8 V, whose endpoint corresponds to Q_{gd} in Figure 8 and Table 2 of the main text.

Table S1. Comparison of gate–drain charge at different voltage-grid resolutions.

Voltage-grid resolution ΔV_{DS} (V)	Conventional SGT Q_{gd} (nC/cm²)	3FPI-SGT Q_{gd} (nC/cm²)
0.5	20.84	23.87
0.2	20.84	23.87
0.1	20.84	23.87
0.05	20.84	23.87
